# Mitigating negative emotions through virtual reality and embodiment

**DOI:** 10.3389/fnhum.2022.916227

**Published:** 2022-08-03

**Authors:** Maria Sansoni, Giovanni Scarzello, Silvia Serino, Elena Groff, Giuseppe Riva

**Affiliations:** ^1^Humane Technology Lab, Università Cattolica del Sacro Cuore, Milan, Italy; ^2^Radiotherapy, Veneto Institute of Oncology - IOV IRCCS, Padua, Italy; ^3^Technology for Neuro-Psychology Lab, Istituto Auxologico, Milan, Italy

**Keywords:** virtual reality, negative emotion, body illusion, body experience, psycho-oncology, emotion regulation, well-being, embodiment

## Abstract

Oncological treatments are responsible for many of the physical changes (aesthetic and functional) associated with cancer. Because of this, cancer patients are at high risk of developing mental health problems. The aim of this study is to propose an innovative Virtual Reality (VR) training that uses a somatic technique (i.e., embodiment) to create a bridge with the bodily dimension of cancer. After undergoing a psycho-educational procedure, a combination of exposure, out-of-body experience, and body swapping will gradually train the patient to cope with cancer-related difficulties, increasing stress tolerance, and patient empowerment. The most engaging step of this advanced form of Stress Inoculation Training is the body swapping experience, which will guide the patient in embodying a resilient cancer patient who is facing similar difficulties. Through the VR ability to simulate the human brain functioning, and the potential of embodiment to hook to the somatic dimension of illness, we expect that once the concepts endured through the patient’s experience of resilience are triggered, the patient will be more prone to implement functional coping strategies in real life, reaching empowerment and adjusting to the post-treatment difficulties. When the scenarios are built and the training tested, our intervention could be used to support patients with different oncological diseases and who are treated in different cancer hospitals, as well as patients with other non-oncological problems (e.g., social anxiety). Future research should focus on using our paradigm for other clinical populations, and supporting cancer patients in coping with different distressing situations.

## Introduction

In 2020, over 19 million people were diagnosed with cancer worldwide (World Health Organization (WHO), 2020). After receiving their diagnosis, the first challenge cancer patients usually face is undergoing treatments, responsible for many of the physical changes associated with this disease. The impact they have on the patient’s body, in the form of aesthetic (e.g., hair loss, scars) and functional changes (e.g., voice changes, urinary incontinence, sexual dysfunction) affects patients’ social life and psychological well-being (Costa et al., 2016). As a result, cancer patients are at a high risk of developing mental health problems during and after treatment: depression (Massie, 2004; Pitman et al., 2018; Tsaras et al., 2018), anxiety (Vickery et al., 2003; Przezdziecki et al., 2013; Trill, 2013; Pitman et al., 2018; Tsaras et al., 2018), post-traumatic stress disorder (Kangas et al., 2005; Moschopoulou et al., 2018), sexual dysfunctions (Rhoten, 2016), sleep disturbances (Trill, 2013), and suicidal risk (Kam et al., 2015). Despite having the goal of healing the patient, treatments can, therefore, negatively affect patients’ quality of life (QoL; Liao et al., 2019). Since reporting poor QoL is associated with an increased risk of mortality for this population (Karvonen-Gutierrez et al., 2008), it is necessary to intervene against any factor (i.e., psychological) that may worsen patients’ well-being, and reduce their chances of survival. Despite the importance of this need, however, treatment toxicity (e.g., pain, fatigue, nausea) impairs patients’ physical functioning (Murphy et al., 2007; Abrahams et al., 2016), creating an impediment to their psychological support: patients feel debilitated, experience severe symptoms, and need medical support or hospitalization (Feliu et al., 2021). In-person therapies are thus difficult to attend, leaving patients exposed to emotional distress. Technologies can facilitate the implementation of psychological treatments overcoming the necessity for the patient to physically attend the meeting (Sirintrapun and Lopez, 2018). For this reason, health care has nowadays witnessed a shift from in-person to technology-based therapies (Richards, 2013). Among all technologies, however, Virtual reality (VR) is the only device capable of offering an immersive experience, as if the person is physically undergoing a real situation. The perception of being present in the situation (i.e., sense of presence; Murphy et al., 2007) allows patients to receive interventions without leaving their room (thus limiting the possible problems of displacement, and rendering therapy accessible to all), and makes them experience in real-time (Riva, 2005) situations otherwise difficult to access, with the same quality of *in vivo* experiences (Riva, 2009). VR facilitates, in fact, the engagement with the task, creating an emotional connection with it (Flavián et al., 2019a), and guaranteeing that when patients interact in the digital environment they cognitively, affectively, and behaviorally invest with the task, as if they were in real life. Along with this, VR also promotes behavioral intentions (i.e., antecedents of actual behaviors that reflect the eagerness of users to carry out particular behaviors; Flavián et al., 2019a), predisposing the individual to pursue in real life what they experienced through VR, and facilitating with that their ability to concretely imagine how the real-world experience (e.g., post-treatment difficulties) would be. These characteristics render VR a promising tool for health care (Riva, 2003; Pensieri and Pennacchini, 2016; Kim and Kim, 2020), and oncological settings (Chirico et al., 2016).

## Current VR Interventions for Cancer Patients

A recent systematic review (Sansoni et al., 2022) investigated the use of VR interventions to improve cancer patients’ well-being and identified two main techniques currently employed by scholars and clinicians to reach this goal: distraction and exposure. VR distraction is used in cancer care to reduce patients’ distress during medical procedures and treatments (e.g., Mohammad and Ahmad, 2019; Gerçeker et al., 2021; Wong et al., 2021; Hundert et al., 2022). Distraction shifts the attentional focus from unpleasant stimuli toward those that are appealing (Schneider and Hood, 2007), taking advantage of the inability of the human cognitive system to process several data at the same time (Kahneman, 1973). An example of VR distraction is the travel session proposed by Niki et al. (2019), used to alleviate the symptoms experienced by cancer patients undergoing palliative care. VR exposure, on the other hand, is mostly employed in psycho-oncology for educational purposes to reduce procedural anxiety (e.g., Tennant et al., 2021; Turrado et al., 2021; Gao et al., 2022), first addressing frightening stimuli, and then putting correcting information into memory (Kaczkurkin and Foa, 2022). Gao et al. (2022) implemented a VR exposure intervention to show cancer patients contents related to the radiotherapy treatment they were about to start.

VR appears as a viable technique for enhancing cancer patients’ well-being, but the strategies currently used to implement VR interventions present the main limitation of placing the patient in a passive role with respect to their own change. VR exposure and distraction promote, in fact, an external Locus of Control (LoC; Rotter, 1966) in that the patient is not responsible for their own change, and has limited control over the situation (Kennedy et al., 1998). Considering that receiving a cancer diagnosis is already associated with feeling powerless (Lin et al., 2020) and hopeless (Meggiolaro et al., 2016; Uslu-Sahan et al., 2019) due to the unexpected and unpredictable nature of the disease (Lin et al., 2020), psycho-oncological VR therapies should assist patients in recovering control over their life (McCarley, 2009) by empowering them. Through empowerment, people achieve mastery over their own life (Rappaport, 1987) and change (Brown et al., 2015; Buddelmeyer and Powdthavee, 2016), improving disease management, health status, medication adherence (Nafradi et al., 2017) and health outcomes (Marimuthu et al., 2022), thus becoming active participants in their treatment (Tsay and Hung, 2004). Being actively engaged reassures, indeed, patients that they can achieve control over their medical condition, contributing to their satisfaction, fulfillment, and well-being (Marimuthu et al., 2022). But how can we render patients active characters of their own oncological journey?

## A New Cognitive Approach to Psycho-Oncology: The Role of Embodiment

A possible answer to the previous question is to support patients in acquiring coping strategies to actively adjust to the emotional distress they experience. We believe that *embodiment* (Kilteni et al., 2012) could have a key role in achieving this goal. Our paradigm will offer a gradual experience of embodiment that will be implemented before oncological treatments (i.e., surgery, chemotherapy, radiotherapy) start. Despite the well-recognized impact of oncological treatments on patients’ psychological well-being (e.g., Kam et al., 2015; Moschopoulou et al., 2018; Pitman et al., 2018), in fact, interventions in psycho-oncology mostly focus on tertiary prevention, with the main goal of improving outcomes, and lessen the negative effects of the disease on patients’ life (e.g., symptom management, minimization of treatment-related complications; Rosberger et al., 2015). However, if cancer patients have a high chance to experience such difficulties, why do psycho-oncological interventions not focus on preventing mental health problems from happening? Our gradual experience of embodiment is an attempt to answer this question, representing for the patient not only a concrete possibility to get ready to face these difficulties (i.e., training component) but also to reduce the probability of post-treatment psychological complications, working as a protective factor (i.e., preventive component). The possibility to propose this training before the beginning of cancer treatments places, indeed, our paradigm in the position of filling the gap that Rosberger and colleagues identified in 2015, therefore moving psycho-oncology from tertiary to primary prevention. By doing that, psycho-oncology will finally avoid treatment-related psychological difficulties from happening (e.g., anxiety, body image, and social concerns), instead of buffering them once already occurred (Rosberger et al., 2015).

Immediately embodying another patient, however, could be too emotionally demanding for the patient, with the risk of exposing the individual to a situation that they have not yet developed resources for, and that they are not ready to face. For this reason, the patient will start going through simple *exposure* to the stressors, will continue with an *out-of-body-experience* to face themselves from an external perspective (Matamala-Gomez et al., 2020), and only at the end, they will switch their body with that of another cancer patient through *body swapping* (Petkova and Ehrsson, 2008; De Oliveira et al., 2016). In this sense, our paradigm will work as an advanced form of Stress Inoculation Training (SIT; Meichenbaum, 1985). Through gradual and controlled exposure to the stressor, SIT prepares individuals for stressful situations by diminishing the likelihood of a negative psychological reaction (Meichenbaum, 2017). Exactly like SIT, our body illusion paradigm will train the patient to face the stressful situations that may arise during their path as a cancer patient and survivor. By giving the possibility to confront the stressor before happening (i.e., pre-treatment), in a controlled, ecological and safe environment, we expect to increase the patient’s stress threshold and reduce their anxiety. Differently from SIT, however, our training will also offer a gradual embodying experience that will help the patient to deeply identify themselves, not only mentally but also with their body, with the experience of a cancer patient who is facing those difficulties. At the origin of the psychological problems that cancer patients report, in fact, there is always an alteration of the physical dimension: thus, we hypothesize that using a somatic technique that involves the body (i.e., embodiment, body swapping) and its perception could be crucial for the intervention to be effective. This approach can provide the patients with the somatic, immersive, all-encompassing, and emotionally engaging experience that they need to prepare themselves for the post-treatment challenges, restructuring their beliefs and learning how to cope with the difficulties while increasing their stress tolerance. By embodying the experience of a cancer patient who successfully copes with social and body image-related difficulties, we expect that the patient will prime concepts related to resilience, subsequently facilitating functional strategies to cope with their cancer experience (Rosenberg et al., 2013).

**Figure 1:**
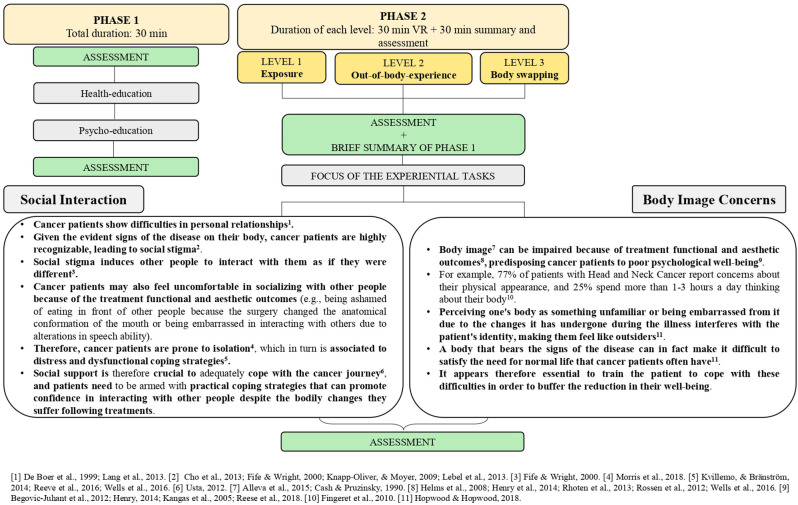
Summary of the intervention, description of the sub-conditions, and duration of each session.

As Figure 1 shows, before starting the experiential tasks, the patient will undergo educational training (phase 1) to equip them with the skills necessary to accomplish the next phase. Firstly, information about oncological treatments, side effects, and difficulties patients usually experience will be provided (i.e., health education). We will then continue with psycho-education focused on providing the patient with basic competencies about how to be resilient. Once phase 1 is completed, we will proceed with phase 2. The experiential training (i.e., phase 2) will give the patient the opportunity to train with three levels of increasingly challenging situations: “*Vicarious Experience and Exposure*”, “*External Perspective and Out-of-body-experience*” and “*Body Swapping*”. Each level will comprehend two sub-conditions to prepare the patient to cope with two specific difficulties they might face following treatments: social interaction and body image concerns (see Figure 1 for more details). To provide ecologic experiences and maximize the sense of presence and embodiment, the features of the scenarios will be those of a real cancer unit (Rauschnabel et al., 2022). In addition to the quality of the virtual experience and to the ecology of the environment, storytelling will have a key role in phase 2 (Gorini et al., 2011; Rosenberg et al., 2013). It will indeed be extremely important to structure the narrative in order to offer a vivid understanding of the situations and experience a sense of immersion and presence in each condition (Gorini et al., 2011). The visuomotor stimuli will be synchronized, and the patients will experience the sub-conditions while wearing a VR headset connected to a camera positioned above their physical body, to mimic a first or third-person perspective depending on the task. The whole training is expected to be implemented as summarized in Figure 1 in the time that occurs from the diagnosis to the beginning of the cancer treatments. At the beginning and at the end of each level patients will undergo an assessment to collect information about the embodiment experience and the clinical variables of interest.

### Level 1: vicarious experience and exposure

**Figure 2:**
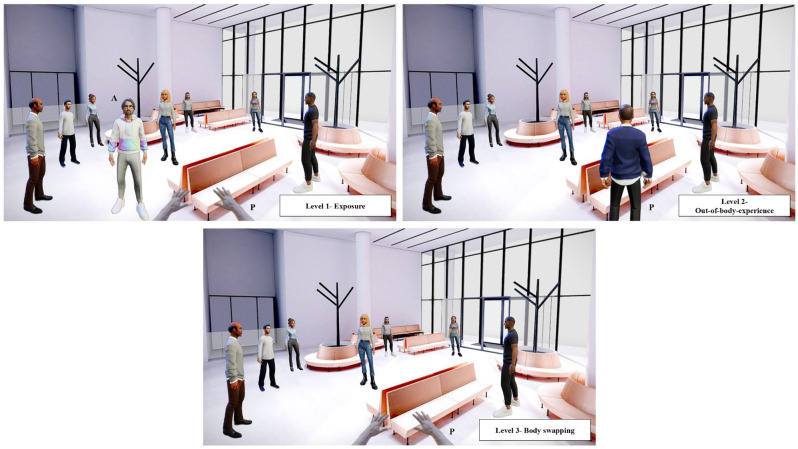
Hypothetical scenarios for levels 1, 2, and 3. In level 1 the patient (P) encourages the virtual avatar (A) to cope with the distress experienced in a social context where other people look at them and make the avatar feel not at ease. In level 2 the patient (P) now experiences the distressing situation from an external perspective. In level 3 the patient (P) embodies the virtual avatar and experiences in first-person the difficulty.

In this level, through the support of the narrative, the participant will be conducted to help another cancer patient (i.e., a virtual avatar) in coping with the difficulties that treatment toxicity involves (i.e., social interaction and body image concerns). The encouragement the patient will provide to the virtual avatar will be recorded and used in the next levels. The patient will have a third-person perspective on the challenges the avatar will face, undergoing a vicarious experience (see Figure 2). The mechanism underlying level 1 is simple exposure: through this step, we expect to reduce the perceived distress by addressing the frightening stimuli (i.e., post-treatment outcomes), and correcting the patient’s beliefs by offering a clear visual idea of the difficulties they may face. In this way, we will support the patient in learning how to cope with the difficulties (i.e., by providing support to the avatar), while emotionally distancing themselves from it (i.e., third-person perspective). Thanks to this, patients will gain the correct understanding of the challenges to face (through exposure), decrease their distress, and will not feel overwhelmed in facing the scenario.

### Level 2: external perspective and out-of-body-experience

Using a well-structured storytelling, the patient will be guided in experiencing the two sub-conditions imaging to be the avatar. To reach this goal we will use an external perspective that reproduces an out-of-body experience view, just by manipulating the position of the camera. The scenario will be the same as level 1; the only difference will be that the patient will see themselves from behind during the training (external perspective; see Figure 2). The patient will listen to their own encouragement (recorded during level 1) while undergoing the two sub-conditions. This new perspective will help the patient to participate in the situation with greater emotional involvement (the person who is undergoing such difficulties is actually them), while facing it from an external perspective.

### Level 3: body swapping

The last level will be the most emotionally engaging: the patient will experience the two sub-conditions by embodying the virtual avatar through body swapping. The patient will switch body and perspective, experiencing the difficulties from a first-person perspective while listening to the encouragement they offered during the previous level, being comforted by their own words. This combination of visual and auditory stimuli through one’s own encouragement has already been used in the study of Falconer et al. (2016), showing effectiveness in reducing depression rates and increasing self-compassion. We expect that being in another patient’s skin might help patients challenge their emotions, and create a deep connection with the experience the avatar is living.

## Discussion and Conclusion

In this work, we identified a new approach to psycho-oncology that aims at reducing patients’ distress by training and preparing them to face the challenges associated with their oncological journey. The innovative component of this paradigm is the gradual introduction of cancer-related stressful situations to the patient through a somatic experience, with the objective of possibly enhancing their coping strategies and resilience. This training is developed to support the patient in gaining functional strategies and empower this clinical population. Our paradigm could potentially reach this goal not only through gradual exposure to the stressful situation as SIT but also by putting each patient in a condition of gradually *learning by doing* (Schank et al., 1999). Learning through one’s own experience intrinsically motivates people to achieve their goals, and to use the tailored skills they acquired when coping with a similar situation (i.e., virtual environment; Schank et al., 1999). In addition to this, since the majority of cancer patients struggle in picturing, and fully understanding how their lives will be once treatments are concluded, the possibility of learning by doing illness-related information could improve patients’ understanding of their cancer journey, reducing the probability of regretting treatment choices (Stryker et al., 2006) and reporting poor health outcomes (Gustafson, 2017). VR is indeed particularly effective in improving understanding, keeping the newly achieved information in memory, and making users experience high engagement in the process and more positive emotions during the knowledge acquisition procedure (Allcoat and von Mühlenen, 2018). The visual stimulation (i.e., virtual scenarios) compensates for a possible lack of imagery (Botella et al., 2004), giving to all patients the same possibility to observe real-life stressors (Murphy et al., 2007), while auditory stimuli, through the storytelling, drive the emotional engagement to the tasks. These components, in addition to the *learning by doing* feature, create and endure memorable experiences that generate a positive cognitive, emotional, social, and physical response in the user (Flavián et al., 2019b), rendering the mix of visual-auditory stimuli with the practical characteristic (i.e., learning by doing) vital ingredients of this intervention.

Along with this, embodying a resilient cancer patient through body swapping could lead to a generalization of what is experienced in VR to the real world: we think that once the concepts endured through the patient’s experience of resilience are triggered, patients will be more prone to implement functional coping strategies in the real life. Therefore, our body illusion paradigm might promote the ability to adapt to stressors and adversities and overcome the negative consequences of risk exposure (i.e., resilience; Herrman et al., 2011). Several authors have already deepened the understanding of the relationship between virtual experience and impact on reality (e.g., Fox and Bailenson, 2009; Hershfield et al., 2011; Rosenberg et al., 2013). According to Rosenberg et al. (2013), embodying certain characteristics in VR not only primes the features observed, but it deeply transforms participants’ self-concept. This assumption is supported by research on the influence of self-concept in mediating the link between concept activation and behaviors (Wyer et al., 2011), as well as by the Narrative Collective-Assimilation hypothesis, stating that an individual undergoing storytelling, mentally becomes a member of the group depicted in the narrative (Gabriel and Young, 2011).

By letting the patient see through the eyes of a person who is living a similar experience, we think that our paradigm could also offer an experience of empathy (Bertrand et al., 2018; Thériault et al., 2021). Using a somatic technique to let people experience the changes they will feel in their bodies post-treatment, we hope to evoke not only cognitive empathy (i.e., the intellectual awareness of one’s emotions and psychological state), but also its bodily correlates (i.e., emotional empathy), matching the emotional perception and the somatic experience of the avatar with those of the patient (Wiederhold, 2020). The embodiment manipulation in this context is indeed aimed at completing the visual stimulation with other bodily information, reaching the somatic dimension of the illness. This sensorial richness deeply affects the virtual experiences undergone by the patients, emotionally engaging them with the task, and facilitating the mental and physical comprehension of the intangible experiences they are undergoing (i.e., difficulties they will face in the future). Embodying experiences creates a sensorial simulation of the events that users have not yet lived, and the virtual experience appears sensorially close to the real one they will face. Because of that, patients feel emotions, perceptions, physiological reactions, and as a response to this bodily engagement, they will feel emotionally, behaviorally, and cognitively different towards the experience (i.e., post-treatment difficulties; Flavián et al., 2019a). This is exceptionally true for the body swapping step: first-person perspective experiences are indeed mentally, emotionally, and physiologically more intense than third-person perspective ones (Slater et al., 2010), and require a mental (e.g., empathizing with the embodied person) and physical (e.g., physiologically) simulation of real-life situations (Preston and De Waal, 2002). The embodiment into an avatar that can freely move and interact within the virtual space (thanks to the combination of data obtained *via* trackers with information from a simulated 3D world) highly mimics brain functioning, which creates embodied simulations of the body in the real environment to represent and anticipate behaviors, thoughts, and emotions (Riva et al., 2018). Body illusions are the maximum representation of this mechanism, and for this reason, we postulate that they are the optimal manipulation to empower patients and to make them experience an engaging simulation of what could become their reality. As VR simulates brain functioning, embodiment resumes the somatic correlates of the oncological disease. Cancer is characterized by a great bodily dimension that affects patients’ psychological experience: embodiment and body swapping propose an equal mechanism, using the body to influence mental states. Due to the somatic nature of the oncological experience and the perception of extraneousness that patients perceive towards their body following treatments, employing a technique that targets the bodily dimension could be crucial. In VR experiences the *sensorialization* of the virtual environment stimulates the individual’s senses as they are in the real world, simulating the spontaneous bodily human functioning. This influences the emotional and behavioral responses, as well as the ease of imagination (Flavián et al., 2021). All these elements are essential in our task to really feel into the virtual patient’s body. The sensory power of embodied technologies has already been used to simulate experiences by creating *pre-experiences* (i.e., in our study, the possibility to face difficulties in advance). The possibility to try ahead before anything happens results in more pleasant actions afterward and in the perception of positive emotions towards the actual experience. Also in this case the key component is the sensory stimulation that VR offers (Flavián et al., 2019a). Since human beings perceive what they have around through their senses, the multi-sensory nature of VR encourages exactly this functioning. Therefore, in accordance with the Theory of Technological Mediation (Ihde, 1990), VR becomes an extension of individuals’ bodies. This assists them in interpreting, perceiving, and interacting with the world they have around (i.e., the virtual world), thus connecting the bodily experiences of the virtual and the real world. This, with the bridge that the use of a somatic technique creates with the bodily nature of cancer, renders our body-oriented paradigm a possibility for the patient’s experiences (i.e., the virtual and the real one) to communicate using a common language: the body. Our paradigm could therefore be considered a *related empowered pre-experience*. According to Flavián et al. (2019b), in these types of experiences the technology itself has a vital role in creating new occurrences within the users’ core experience (i.e., undergoing cancer treatment-related difficulties; Flavián et al., 2019b). In our study, the experience of training patients for facing future difficulties would not be possible without VR, in that for the intervention to work the patient needs to face the difficulties before they actually happen. VR is thus the medium we have to help patients to visualize, and feel on their bodies these experiences as if they are real. However, to disentangle if the embodiment is what renders this VR experience effective, it would be essential to preliminary compare a group of patients that will be subjected to embodiment and body illusions, to patients who will simply be inserted in a random first-person virtual avatar to test for the Proteus Effect (Yee and Bailenson, 2007). Indeed, being placed in a first-person avatar leads to the assumption of values and psychological features of the avatar itself, even in absence of any embodiment protocol (Yee and Bailenson, 2007).

In conclusion, this training represents an innovative approach to psycho-oncology in that, differently from previous interventions (Sansoni et al., 2022), it proposes an active task able to empower patients (the primary goal of the World Health Organization in the “2020 health program”; Tartaglione et al., 2018), it involves the body, promoting an approach that encourages the mind-body relationship (Riva et al., 2017), and it updates a technique, the Stress Inoculation Training, well-known to be effective but that has never been renewed since its creation (Meichenbaum, 1985). Furthermore, this training applies body swapping to a totally new field (i.e., oncology), and it aims at preventing psychological problems before they happen instead of treating them once already emerged, as psycho-oncological interventions usually do (Rosberger et al., 2015). In addition to those just mentioned, a further contribution of our training is also to promote patient-centered medicine, empowering patients and rendering them active characters in their own health management (Tartaglione et al., 2018). Nowadays, “patient-centeredness” is indeed a core dimension of healthcare, and an essential feature for a 360-degree improvement of the quality of the health system (Tartaglione et al., 2018).

## Practical Implications

The main practical implication of our paradigm in cancer care is that when the scenarios are built and the training tested, our intervention could be used to support patients with different oncological diseases, and who are treated in different cancer hospitals. Since social and body image concerns are indeed frequent in all the different oncological conditions, it would be sufficient to modify the storytelling in order to tailor the scenarios for specific oncological experiences (e.g., for example, tailoring the narrative to the speech difficulties of patients with head and neck cancer undergoing a total laryngectomy in communicating with others), or on the contrary keeping it more generic in order to give the opportunity to all cancer patients to find their own, personal meaning within the same experience. This is expected to reduce the costs of cancer care (e.g., fewer medications, greater adherence to treatments) and the need for additional human resources, resulting in benefits not only for patients but also for the institutes that will adopt this paradigm (Simpson et al., 2001). In addition to this, since we are proposing an advanced form of SIT, our paradigm could be broadly used in several clinical settings, not necessarily related to cancer care. For example, the same scenarios we employed could be used for social anxiety. Socially anxious individuals experience discomfort in social situations, where their social status is threatened, and they worry about their own or others’ unfavorable opinions. In such contexts, individuals may report bodily experiences (i.e., sweating, shaking, and blushing) that are particularly feared by socially anxious individuals, in that they are highly visible, and represent a source of embarrassment for them. Even if the somatic experience (e.g., blushing) does not occur, socially anxious people are so worried that this may happen that they avoid any social setting (Drummond et al., 2020). In this sense, both the social scenario, where participants interact with others and the body concerns experience of our training, where participants challenge themselves with difficulties related to their bodies, may be relevant for socially anxious people. Our virtual training can thus support them in gradually acquiring confidence in such situations while gaining functional coping skills and empowerment, through a somatic experience that can contact exactly the bodily components of social anxiety. Also, in this case, the narrative can be modified in order to specifically tailor the experience to what is perceived as distressing for socially anxious individuals. Future research should thus focus on using our paradigm with other clinical populations (oncological and non-oncological), supporting cancer patients (and not only) in coping with different distressing situations (e.g., chronic symptoms management). If the intervention will be found effective, future research should focus also on analyzing the short and long-term effects of this approach, to understand the extent of its benefits.

A key objective in the promotion of well-being is, in fact, to deliver interventions that are affordable and available to everyone, and with this paradigm, that will be accessible to everybody and easily reproducible both in oncological and non-oncological settings through cardboards and cheap VR headsets, we hope to accomplish this goal. With this training, that was born for cancer patients but that could easily be adapted to other situations, we truly believe that we can have a broad impact on health care and support individuals with different physical and psychological conditions.

## Data Availability Statement

The original contributions presented in the study are included in the article, further inquiries can be directed to the corresponding author.

## Author Contributions

MS designed and planned the study with the support of GR. MS wrote the first draft of the manuscript and then SS and GR contributed to the final version, supervising the description of the parts of the manuscript, as well as the rationale and the scientific contributions. GS and EG supervised the oncological contents. All authors contributed to the article and approved the submitted version.

## Funding

This research was funded by Italian Ministry of Health Ricerca Corrente 2022.
